# Ambient Particulate Matter Air Pollution in Mpererwe District, Kampala, Uganda: A Pilot Study

**DOI:** 10.1155/2014/763934

**Published:** 2014-02-17

**Authors:** Stephan Schwander, Clement D. Okello, Juergen Freers, Judith C. Chow, John G. Watson, Melody Corry, Qingyu Meng

**Affiliations:** ^1^Department of Environmental and Occupational Health and Center for Global Public Health, Rutgers School of Public Health, Piscataway, NJ, 08854, USA; ^2^Department of Medicine, Makerere University College of Health Sciences, Makerere University School, Kampala, Uganda; ^3^Division of Atmospheric Sciences, Desert Research Institute, Reno, NV 89512, USA; ^4^Division of Global Affairs, Rutgers the State University of New Jersey, Newark, NJ 07102, USA

## Abstract

Air quality in Kampala, the capital of Uganda, has deteriorated significantly in the past two decades. We made spot measurements in Mpererwe district for airborne particulate matter PM_2.5_ (fine particles) and coarse particles. PM was collected on Teflon-membrane filters and analyzed for mass, 51 elements, 3 anions, and 5 cations. Both fine and coarse particle concentrations were above 100 µg/m^3^ in all the samples collected. Markers for crustal/soil (e.g., Si and Al) were the most abundant in the PM_2.5_ fraction, followed by primary combustion products from biomass burning and incinerator emissions (e.g., K and Cl). Over 90% of the measured PM_2.5_ mass can be explained by crustal species (41% and 59%) and carbonaceous aerosol (33%–55%). Crustal elements dominated the coarse particles collected from Kampala. The results of this pilot study are indicative of unhealthy air and suggest that exposure to ambient air in Kampala may increase the burden of environmentally induced cardiovascular, metabolic, and respiratory diseases including infections. Greater awareness and more extensive research are required to confirm our findings, to identify personal exposure and pollution sources, and to develop air quality management plans and policies to protect public health.

## 1. Introduction

Air pollution is an increasing problem in urban dwellings worldwide. The World Health Organization (WHO) ranks indoor air pollution (from biomass fuel combustion) and urban outdoor air pollution 10th and 14th, respectively, among 19 leading risk factors for global mortality [1]. There is indisputable epidemiologic evidence that air pollution exposure in humans increases not only the risk of respiratory conditions such as asthma and respiratory tract infections but also cardiovascular disease and metabolic diseases such as diabetes mellitus [2, 3]. In the developing world, air pollution presents a particular threat to the health of populations in cities that are growing in a fast, poorly planned, and unregulated manner [4, 5].

This is the case in Kampala, Uganda, where air quality has deteriorated significantly during the past two decades in parallel with the fast-paced economic development. Kampala is the capital of Uganda in East Africa, a country with a rapidly increasing population and expanding economic growth. With a growing middle class, Uganda, and specifically Kampala, has seen fast expanding vehicular traffic comprised of imported second hand and reconditioned cars, aging, exhaust-unregulated cars, trucks, buses, and motorcycles. Furthermore, the national economic strategy gives priority to the industrial and manufacturing sectors and has promoted and realized a rise in cottage and other larger industries within the city. In addition to the ever growing road traffic and unregulated industrial emissions, waste burning by individuals to manage uncollected waste, also contributes to the deterioration of air quality in Kampala.

However, reports on air pollution in Kampala are primarily anecdotal [6–8]. An ongoing study shows an increase of ischemic heart disease from virtually absent up to the end of the 1990s to more than 10% of all admissions on the Mulago University Hospital cardiology ward in 2011. As part of an effort to preliminarily characterize airborne particulate matter (PM) within Kampala, we made spot measurements of ambient PM in a typical bustling, low-income, mixed commercial, and residential section of the city (Mpererwe District). This paper provides a summary of the findings.

## 2. Methods

**Figure 1:**
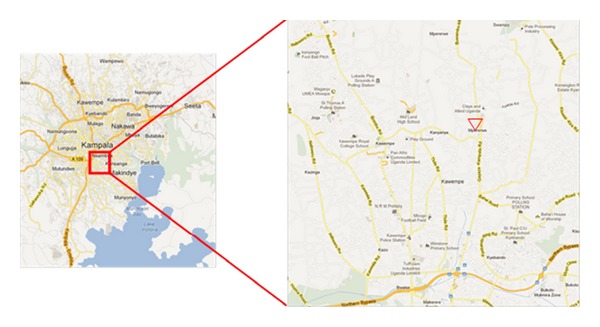
Map showing Kampala with detail of the sampling area. Sampling location indicated with red triangle. Source: Google maps (https://maps.google.com/maps?hl=en&tab=wl).

To characterize the content of air pollution PM, we made spot measurements in Mpererwe District, Kawempe South Division, at Kalerwe Market, 80 meters east from Gayaza Kampala Road in Kampala City (Figure 1—Map). Employing a Sioutas Personal Cascade Sampler (SKC Inc., Eighty Four, PA, USA) with a flow rate of 9 liters per minute, we collected fine particles (PM_2.5_, or particles with aerodynamic diameters less than 2.5 *μ*m) and coarse particles (PM_7-2.5_) (this fraction contains total suspended particles with aerodynamic diameters greater than 2.5 *μ*m) during two 24-hour sampling periods (6:30 p.m. to 6:30 p.m., Local Standard Time [LST]) between December 31st, 2012, and January 1st, 2013, and between January 2nd, 2013, and January 3rd, 2013. PM were collected on Teflon-membrane filters (37 mm, 2 mm pore, Pallflex Gelman Scientific, Ann Arbor, MI, USA). The personal PM monitor as well as a Leland Legacy Pump (SKC Inc., Eighty Four, PA, USA) were installed 3 meters above ground level, on the outside wall of a single story structure under a 50 cm protruding metal sheet roof.

PM_2.5_ and PM_coarse_ samples were removed from the monitors at the end of each sampling period, stored in plastic petri dishes, sealed and transferred to the USA for further analysis. Filters were equilibrated under a temperature (21.5 ± 1.5°C) and relative humidity (35 ± 5%) controlled environment before gravimetric analysis following the US Environmental Protection Agency (EPA) protocol at the Environmental and Occupational Health Sciences Institute (EOHSI) in New Jersey. PM_2.5_ samples were submitted for elemental analysis by X-ray fluorescence (XRF) including 51 elements from Na to U (see Table 1 and [10]). Three anions (Cl^−^, NO_3_^−^, and SO_4_^2−^) and five cations (NH^+^, Na^+^, Mg^2+^, Ca^2+^, and K^+^) were measured for PM_2.5_ by ion chromatography (IC) [11]. Particle loadings from the personal PM monitor are centered on a slit, so only 18 elements were analyzed by inductively coupled plasma-mass spectrometry (ICP-MS).

## 3. Results

Based on the PM mass, fine and coarse particle concentrations were 104.9 *μ*g/m^3^ and 132.7 *μ*g/m^3^ for the sample from December 31, 2012 and 103.7 *μ*g/m^3^ and 208.1 *μ*g/m^3^ for the sample from January 2, 2013, respectively.

**Figure 2:**
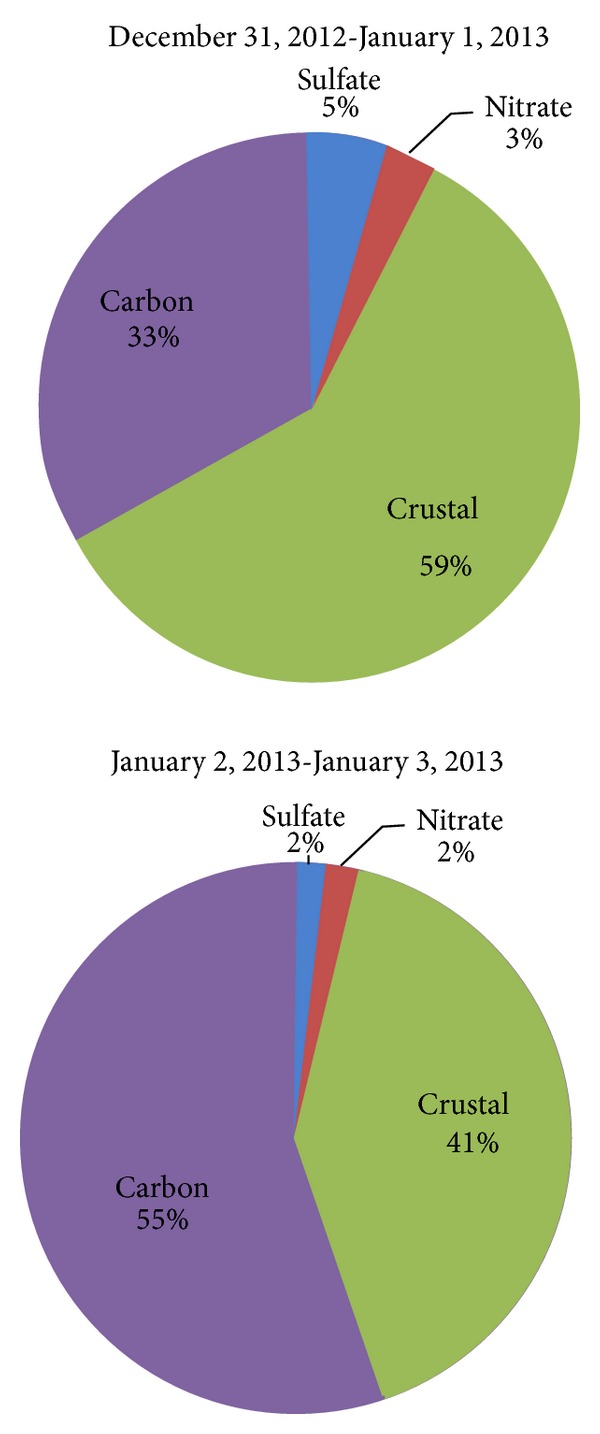
Twenty-four-hour average PM_2.5_ compositions of major components based on mass reconstruction, where fine soil = 2.2Al + 2.49Si + 1.63Ca + 2.42Fe + 1.94Ti [9]. Ammonium (NH_4_^+^) concentrations are low, so only sulfate and nitrate are included. The remaining PM_2.5_ mass is assumed to be carbon.

PM_2.5_ mass concentrations and chemical compositions are listed in Table 2, which includes typical marker species representing major PM_2.5_ sources, such as soil (Al, Si, Ca, Fe, and Ti), oil combustion (V and Ni), biomass combustion (K^+^), traffic (Pb and Zn), incinerator (Cl), and secondary PM (SO_4_^2−^). A full list of all measured PM_2.5_ species is in Table 1. The relative abundance of major PM_2.5_ species is similar across the two sampling days. Markers for crustal/soil are the most abundant, followed by primary combustion sources, such as biomass burning and incinerator emissions. Elevated water-soluble to total potassium ratio (K^+^/K = 0.91) for both PM_2.5_ samples confirms an abundance of biomass burning during the sampling period [12]. An exploratory PM_2.5_ mass reconstruction was conducted based on major species measured, that is, SO_4_^2−^, NO_3_^−^, Al, Si, Ca, Fe, and Ti, and closing the mass balance by assuming the remaining mass was contributed by carbon species [13].  Figure 2 illustrates the percent contributions of SO_4_^2−^, NO_3_^−^, crustal sources, and carbon. Over 90% of measured PM_2.5_ mass can be explained by crustal species and carbonaceous aerosol.  Table 3 shows the elemental concentrations measured for PM_coarse_ samples. Abundant (>200 ng/m^3^) crustal species (e.g., Al, Ca, and Fe) are found with large sample-to-sample variations.

The meteorological conditions during the sampling period are presented in Table 4. Mean temperatures across the four sampling dates ranged from 21.7°C to 24.4°C, with dew point temperatures between 18.9 and 20.0°C and mean wind speeds between 6.4 and 8.1 Km/hr.

## 4. Discussion

**Figure 3:**
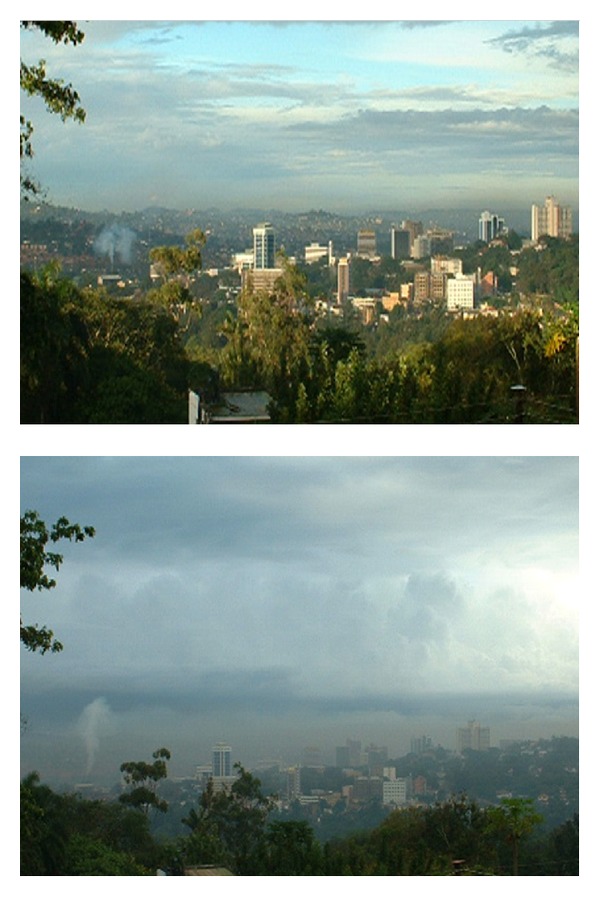
Views of the center of Kampala on a clear day (top) and a day with extensive air pollution (bottom).

To the best of our knowledge, there is no known data/sample collection and public information available on type and concentrations of particulate matter in Kampala's air and studies of the associated human health impacts are extremely limited. There is clear evidence that air pollution in Kampala has increased in the last decade (Figure 3) and that awareness about this problem is mounting [6–8]. The limited findings of the current study clearly support these observations and indicate that exposure to ambient air in Kampala may raise public health concerns such as the burden of cardiovascular and respiratory diseases. PM_2.5_ mass concentrations observed in Kampala are three and four times higher than the US 24-hr PM_2.5_ National Ambient Air Quality Standards (NAAQS; 35 *μ*g/m^3^) and the WHO air quality guidelines (25 *μ*g/m^3^), respectively. This implies that air in Kampala could cause a suite of adverse health effects, especially in susceptible populations such as children and the elderly [3]. The observed PM_2.5_ levels in Kampala are comparable to the PM_2.5_ levels in other megacities in the developing world. For example, the mean PM_2.5_ concentration in Beijing one month before the Olympic Games was 98.9 ± 14.7 *μ*g/m^3^ [14]; Cao et al. (2013) reported 24 hr PM_2.5_ concentrations ranged from 74.2 ± 27.2 *μ*g/m^3^ to 356.3 ± 118.4 *μ*g/m^3^ across 14 Chinese cities in the winter of 2003 and ranged from 27.3 ± 11.0 *μ*g/m^3^ to 131.6 ± 28.0 *μ*g/m^3^ in the summer of 2003 [15]. Kulshrestha et al. (2009) reported that the concentration of PM_2.5_ in Agra, India ranged from 22.8 *μ*g/m^3^ to 215.1 *μ*g/m^3^ with a mean of 104.9 ± 47.1 *μ*g/m^3^ and a standard deviation of 47.1 *μ*g/m^3^ [16]. In a measurement campaign, Chow et al. (2002) reported that the mean and the maximum PM_2.5_ mass concentrations in Mexico City were 35.2 and 183.7 *μ*g/m^3^, respectively [17]. It should be pointed out that the average concentrations from world cities mentioned above and in the literature were the monthly, seasonal, or annual average of measured daily PM_2.5_ concentrations. Therefore, caution needs to be exercised when comparing our results and the data presented in the literature.

Coarse particles collected in Kampala were primarily composed of crustal elements (i.e., Al, Ca, and Fe), indicating soil dust as the major source. Unlike PM_2.5_, coarse particles are formed in the atmosphere through mechanical forces, for example, the resuspension of road dust. Indeed, many unpaved road sections around the sampling area were observed by the authors. Other activities, such as agriculture, can also contribute to airborne coarse particles. These findings warrant further studies to examine the air pollutant source contributions.

Various anthropogenic sources appear to contribute to the elevated PM_2.5_ levels in Kampala. In 2011, the Uganda Revenue Authority (URA) estimated there were 635,656 vehicles in Uganda and about 50% of those were operating in greater Kampala [18]. Between 4,000 and 5,000 vehicles enter the country monthly from various countries [8, 19]. These vehicles are mostly second-hand and purchased reconditioned from Japan. To date, there are no age restrictions and requirements for emission restrictions in place that exist in industrialized countries [20, 21]. According to the Uganda National Environment Management Authority (NEMA), all the resulting daily traffic jams result not only in loss of valuable health and time but also lead to a loss of about 500 million Uganda Shillings (about US $200,000) in burnt fuel; that is, about 140,000 liters of fuel are burnt by idling cars every day [18].

Furthermore, the Bank of Uganda estimates that by 2003 there were 800,000 small scale enterprises in the entire country of Uganda, providing employment and income generation opportunities to low income sectors of the economy, but at the same time, potentially contributing to air pollution. In addition, there is no mandatory garbage collecting system in Kampala, Uganda. The majority of inhabitants has been and continues to simply burn their waste which includes a lot of plastics and results in toxic smoke.

The increased source emissions of PM_2.5_ may result from the rapid urbanization. The National Development Plan 2010–2015 estimates that Uganda's population could double to nearly 61 million in about 22 years [22]. The city has spread from the original 7 hills it sat on at independence in 1962, to approximately 50 now. City planners at the time planned urban infrastructure for approximately 500,000 residents. Today, Kampala has approximately 1,819,200 inhabitants (extrapolated from a population of 1,420,200 in 2008 and a population of 1,659,600 on June 16th 2011) and an additional estimated 2.5 million commuters from outside populating the roads of the city every day. Ever since independence no major infrastructural changes were initiated to accommodate this increase. The City's governing bodies are unable to cope with this massive population influx and the resulting consequences such as overcrowding, uncollected garbage, increased individual motor-vehicle traffic in absence of an organized public transport system, mostly on unpaved roads and uncontrolled informal markets, and small industries.

Given the evidence of abundant vehicle engine emissions and biomass burning in the PM samples from this study, future studies will have to include analysis of organic and elemental carbon as well as carbon speciation to further understand the source attribution and its potential health impacts. The current study is limited by a small sample size, limited PM speciation, assessment of PM at one location only, and by lack of seasonal assessments. The results of this pilot study are indicative of unhealthy air in Kampala and suggest that a large number of people may be threatened by cerebro- and cardiovascular as well as respiratory tract diseases and even diabetes mellitus resulting from air pollution exposure. Greater awareness and more extensive research on air pollution in Kampala are required to confirm our findings, to identify pollution sources that contribute to personal exposure, and to develop air quality management plans and policies to improve public health.

## Figures and Tables

**Table 1:** Concentrations of all measured PM_2.5_ species (ng/m^3^).

PM_2.5_ species	Analytical method^a^	December 31, 2012-January 1, 2013	January 2, 2013-January 3, 2013	Limit of detection
Na	XRF	0	0.0	0.0
Mg	XRF	0	0.0	23.7
Al	XRF	5451	4097.6	17.6
Si	XRF	9757	6739.5	0.9
P	XRF	30	16.0	2.1
S	XRF	1312	632.7	0.0
Cl	XRF	1959	1047.5	0.1
K	XRF	2969	1976.1	0.4
Ca	XRF	3357	1628.8	7.2
Sc	XRF	0	0.0	5.4
Ti	XRF	779	491.7	0.4
V	XRF	10	7.9	0.0
Cr	XRF	16	8.9	0.2
Mn	XRF	158	109.8	0.4
Fe	XRF	7616	5400.2	4.5
Co	XRF	0	0.0	0.7
Ni	XRF	3	11.1	0.7
Cu	XRF	16	10.3	0.4
Zn	XRF	90	79.4	0.4
Ga	XRF	0	0.0	0.0
As	XRF	8	2.3	0.0
Se	XRF	0	0.7	0.7
Br	XRF	41	28.3	0.7
Rb	XRF	15	7.3	0.2
Sr	XRF	35	104.2	0.9
Y	XRF	6	2.5	0.3
Zr	XRF	56	29.9	1.9
Nb	XRF	0	0.0	0.7
Mo	XRF	0	0.0	0.7
Pd	XRF	0	0.0	4.1
Ag	XRF	0	0.0	0.7
Cd	XRF	0	0.0	0.0
In	XRF	0	2.2	2.6
Sn	XRF	0	0.0	1.0
Sb	XRF	0	0.0	8.3
Cs	XRF	4	0.0	0.0
Ba	XRF	0	0.0	12.3
La	XRF	2	0.0	17.3
Ce	XRF	0	0.0	34.4
Sm	XRF	37	1.9	65.4
Eu	XRF	0	0.0	30.9
Tb	XRF	35	17.1	68.3
Hf	XRF	0	0.0	0.0
Ta	XRF	0	0.0	0.0
W	XRF	0	0.0	1.7
Ir	XRF	0	0.0	0.0
Au	XRF	0	0.0	1.7
Hg	XRF	0	0.0	0.0
Tl	XRF	0	0.0	0.4
Pb	XRF	23	18.6	1.2
U	XRF	0	0.0	0.0
Cl^−^	IC	1984	1160.5	0.0
NO_3_^−^	IC	2547	1502.4	72.6
SO_4_^2−^	IC	3542	1381.7	8.9
Na^+^	IC	445	198.7	4.8
NH_4_^+^	IC	141	97.6	0.0
K^+^	IC	2705	1803.4	78.7
Ca^2+^	IC	2409	959.2	2.3
Mg^2+^	IC	223	125.8	0.0

^a^XRF: X-ray fluorescence; IC: Ion chromatography.

**Table 2:** PM_2.5_ mass (*µ*g/m^3^) and species concentrations (ng/m^3^).

PM_2.5_ species	December 31, 2012-January 1, 2013	January 2, 2013-January 3, 2013
Mass	104.9	103.7
Cl^−^	1983.5	1160.5
NO_3_^−^	2547.1	1502.4
SO_4_^2−^	3542.0	1381.7
Na^+^	445.4	198.7
NH_4_^+^	141.3	97.6
K^+^	2705.4	1803.4
Ca^2+^	2409.5	959.2
Mg^2+^	222.5	125.8
Al	5450.9	4097.6
Si	9756.6	6739.5
Ca	3356.8	1628.8
Ti	779.2	491.7
Fe	7616.0	5400.2
V	10.3	7.9
Ni	3.2	11.1
K	2969.2	1976.1
Se	0.0	0.7
Cl	1958.7	1047.5
Br	40.9	28.3
As	7.8	2.3
Pb	22.9	18.6
Zn	90.0	79.4
S	1311.7	632.7

**Table 3:** PM_coarse_ mass (*µ*g/m^3^) and species concentrations (ng/m^3^).

Coarse PM species	December 31, 2012-January 1, 2013	January 2, 2013-January 3, 2013
Mass	132.7	208.1
Mg	31.4	386.5
Al	694.8	6639.4
Ca	248.8	2324.4
V	1.2	12.6
Cr	5.1	12.1
Mn	9.6	95.1
Fe	570.3	5163.0
Ni	0.6	4.0
Cu	2.2	7.4
Zn	5.9	35.8
Mo	0.1	0.7
Ag	0.0	0.1
Cd	0.0	0.0
Sn	0.0	0.6
Ba	3.3	28.9
Ce	1.6	15.0
Hg	0.6	3.6
Pb	0.8	7.3

**Table 4:** Meteorological conditions during sampling.

Meteorological parameters	December 31, 2012	January 1, 2013	January 2, 2013	January 3, 2013
Mean temperature, °C	22.2	21.7	24.4	23.3
Maximum temperature, °C	25.0	26.1	27.8	26.7
Minimum temperature, °C	18.9	17.2	20.6	20.6
Dew point temperature, °C	18.9	19.4	20.0	20.0
Mean wind speed, Km/hr	8.1	6.4	8.1	8.1
Maximum wind speed, Km/hr	16.1	16.1	19.3	19.3
